# Natural variation in the expression of *ORGANIC CATION TRANSPORTER 1* affects root length responses to cadaverine in *Arabidopsis*

**DOI:** 10.1093/jxb/eru444

**Published:** 2014-11-16

**Authors:** Allison K. Strohm, Laura M. Vaughn, Patrick H. Masson

**Affiliations:** Laboratory of Genetics, University of Wisconsin Madison, 425G Henry Mall, Madison, WI 53706, USA

**Keywords:** *Arabidopsis*, cadaverine, natural variation, polyamines, roots, transporter.

## Abstract

Polyamines, including cadaverine, are organic cations that affect numerous biological processes including transcription, translation, cell signalling, and ion channel activity. They often function in biotic and abiotic stress responses in plants. Because little is known about how plants respond to cadaverine, a quantitative natural variation approach was used to identify genetic factors that contribute to this response. Here it is shown that *Arabidopsis thaliana* accessions have varying root length responses to exogenous cadaverine: Cape Verde Islands (Cvi) was one of the most resistant accessions tested, whereas Landsberg *erecta* (L*er*) was one of the most sensitive. Recombinant inbred lines, near isogenic lines, and a microarray were used to show that variation in *ORGANIC CATION TRANSPORTER 1* (*OCT1*) is at least partially responsible for this difference. *OCT1* expression was higher in Cvi than in L*er*, and *oct1* mutants were more sensitive to cadaverine than wild-type plants. In *oct1* mutants transformed with an ectopic copy of *OCT1* originating from either Cvi or L*er*, the expression level of the transgene, not its accession, correlated with the cadaverine response. These results suggest that decreased *OCT1* expression confers cadaverine sensitivity in some accessions.

## Highlight

How the polyamine cadaverine alters plant development is poorly understood. Here it is shown that natural variation in *ORGANIC CATION TRANSPORTER 1* affects root length sensitivity to exogenous cadaverine.

## Introduction

Polyamines are organic cations that are prevalent in both prokaryotes and eukaryotes. Putrescine, spermidine, and spermine are the most common polyamines in plants, although thermospermine and cadaverine have also been found in many species including *Arabidopsis thaliana* (Shevyakova *et al.*, 2001; Knott *et al.*, 2007). Polyamines exist both as free molecules and as conjugates to other small molecules, mainly hydroxycinnamic acids, or proteins (Bagni and Tassoni, 2001). Both free and conjugated polyamines are important for cellular processes including cell growth, cell division, transcription, translation, and cell death. Their positive charges allow for interactions with nucleic acids, lipids, and proteins, which affect the activities and stabilities of these molecules (for a review, see Hussain *et al.*, 2011).

Polyamines accumulate in certain plant tissues under many abiotic stresses including drought, salt, osmotic, and temperature stress, which all result in metabolic reprogramming and modifications to growth (Cuevas *et al.*, 2008; Alet *et al.*, 2011; Wang *et al.*, 2011). Polyamines may protect against these stresses in several ways, including by altering gene expression, increasing the activities of antioxidant enzymes, and altering the cellular accumulation of ions (Liu *et al.*, 2000; Takahashi *et al.*, 2003; Tang and Newton, 2005). They may also function as signals that regulate cross-talk between hormonal pathways during stress responses (Obata and Fernie, 2012). Though essential for proper development, and beneficial at higher levels during stress, catabolism of polyamines produces H_2_O_2_ (Tisi *et al.*, 2011). Although it is toxic, H_2_O_2_ also diffuses to nearby tissues where it activates various responses (Neill, 2002). To balance these effects, polyamine synthesis, degradation, localization, and conjugation must be tightly controlled.

Five potential *Arabidopsis* POLYAMINE UPTAKE TRANSPORTER (PUT) proteins were identified based on sequence similarity to known polyamine transporters in *Leishmania major* and *Trympanosoma cruzi* (Mulangi *et al*., 2012*a*, *b*). When expressed in the polyamine uptake-deficient *Δagp2 Saccharomyces cerevisiae* mutant (Aouida *et al.*, 2005), these proteins partially restored sensitivity to the polyamine analogue paraquat as well as to putrescine and spermidine. They also partially restored putrescine and spermidine uptake into the cells (Mulangi *et al.*, 2012*a*). PAR1/PUT2 has subsequently been shown to localize to the Golgi where it is required for the subcellular localization of paraquat in the chloroplast (Li *et al.*, 2013). *RMV1*/*PUT3* has been shown to be plasma membrane associated and to function in paraquat, putrescine, spermidine, and spermine uptake in *Arabidopsis* (Fujita *et al.*, 2012). However, cadaverine transport by these proteins was not tested.

Less is known about cadaverine compared with the other polyamines. It is synthesized from lysine and therefore not directly derived from the polyamine putrescine, unlike spermidine, spermine, and thermospermine. Cadaverine is produced by some soil bacteria and yeast, possibly contributing to rhizosphere–root signalling (Perrig *et al.*, 2007; Cloete *et al.*, 2009). Exogenous cadaverine affects root development in several ways: it increases adventitious root growth in pine trees, lateral root formation in soybeans, and root weight in rice (Gamarnik and Frydman, 1991; Niemi *et al.*, 2002; Cassán *et al.*, 2009), whereas it shortens primary roots in soybeans (Gamarnik and Frydman, 1991).

Cadaverine levels and localization change upon exposure to stress. This molecule accumulates dramatically upon oxidative stress in rape leaves and upon drought stress in peppers (Aziz *et al.*, 1997; Sziderics *et al.*, 2010). In the ice plant, salt stress causes cadaverine to accumulate in true leaves (Kuznetsov *et al.*, 2007), and heat shock stimulates cadaverine transport away from the heated tissue (Shevyakova *et al.*, 2001). Cadaverine also confers protection against stress. It induces stomatal closure, a common stress response, in fava beans (Liu *et al.*, 2000), and it prevents decreases in root and shoot weight upon oxidative stress in rice (Cassán *et al.*, 2009).

Despite its profound impacts on overall cellular homeostasis and plant growth under stressful conditions, the underlying mechanisms that explain how cadaverine alters plant growth and development are still unknown. To address this gap, natural variation in root length sensitivity to exogenous cadaverine was examined, and *ORGANIC CATION TRANSPORTER 1* (*OCT1*, *AT1G73220*) was identified as an important quantitative trait locus (QTL) involved in this response.

## Materials and methods

### Plant materials

QTL analysis used the following stocks from the Arabidopsis Biological Resource Center (Columbus, OH, USA): Cape Verde Islands (Cvi; CS8580), Landsberg *erecta*-2 L*er*-2; CS8581), and a population of Cvi/L*er* recombinant inbred lines (RILs; CS22000). This population consists of 162 individual RILs that were genotyped at 293 marker loci (Alonso-Blanco *et al.*, 1998). The near isogenic lines (NILs) LCN1-12, -15, -14, -21, -22, and -28 (N717058, N17060, N17061, N17067, N17068, and N17074) (Keurentjes *et al.*, 2007) were obtained from the Nottingham Arabidopsis Stock Center (Nottingham, UK). An-1 (CS22626), Bs-1 (CS6627), Knox-18 (CS22567), Sq-8 (CS22601), Ull2-3 (CS22587), and Wei-0 (CS22622) seed came from the Arabidopsis Biological Resource Center. The *oct1-1* mutant and *oct1-1* [35S_pro_:OCT1^WS^] transgenic lines were provided by Christine Lelandais-Briere (Lelandais-Brière *et al.*, 2007).

### Growth conditions

The seeds were sterilized by washing them with 95% ethanol. Except where noted in the text, they were plated on half-strength buffered Linsmaier and Skoog medium containing micro- and macronutrients, vitamins, and 1.5% sucrose (Caisson Laboratories, North Logan, UT, USA) that was supplemented with 1.5% agar type E (Sigma-Aldrich, St. Louis, MO, USA) and polyamines (Sigma-Aldrich) as required. The seedlings were stratified for 2–8 d and grown in either a Conviron TC16 growth chamber or an Enconair (now BioChambers, Winnipeg, Manitoba, Canada) Controller 6000 growth chamber for each experiment. Both chambers were set at 22 °C and a 16h light/8h dark cycle, and the light intensity was 50–70 μmol m^–2^ s^–1^ and provided by cool-white fluorescent bulbs (Grainger, Lake Forest, IL, USA).

### Root length measurements

Seedling images were taken from the backs of the plates using a scanner (Epson, Suwa, Nagano Prefecture, Japan). Root length was measured using the segmented line tool in NIH Image (publically available at http://rsb.info.nih.gov/nih-image/) or the Neuron J plugin (Meijering *et al.*, 2004) for Image J (Schneider *et al.*, 2012).

### Microscopy

To calculate cell length, the cell walls were stained by transferring the seedlings to 10 μg ml^–1^ propidium iodide (Sigma-Aldrich) for ~20min. Imaging was conducted using a Zeiss LSM 510 Meta Confocal microscope (Zeiss, Thornwood, NY, USA), and cell length measurements were performed in Adobe Photoshop.

### QTL mapping

Plants were grown at a 30 ° tilt for 6 d on media with or without cadaverine. A concentration of 50 μM cadaverine was used in trial 1, and 100 μM cadaverine was used in trial 2. The mean root length value on medium containing cadaverine was subtracted from the mean value on control medium and then divided by the mean on control medium. This number was subtracted from 1, and this ratio, along with genotypic data for each RIL (Alonso-Blanco *et al.*, 1998), was entered into WinQTL Cartographer version 2.5 (Wang *et al.*, 2010) and the r/qtl package in R (http://www.r-project.org). For WinQTL Cartographer, maps were created using the Kosambi map function. During composite interval mapping, the model incorporated a 0.5 step rate with a 1.0 cM window size and 10 control markers. LOD thresholds were determined by 1000 permutations. In r/qtl, the function ‘scanone’ was used for interval mapping, and ‘scantwo’ was used to generate the two-dimensional plot. Significance levels were determined by 1000 permutations, implementing the EM algorithm. Fine mapping of the NILs was performed using standard methods and the primers listed in Supplementary Table S1 available at *JXB* online.

### RT–PCR and qRT-PCR

Approximately 24 seedlings were grown at a 30 ° backward tilt at staggered times for each biological replicate on medium containing no cadaverine unless otherwise indicated. Whole roots from 7-day-old seedlings were dissected and immediately frozen in liquid nitrogen. RNA was prepared using an RNeasy Plant Mini Kit (QIAGEN, Venlo, The Netherlands) and stored at –80 ºC until use, typically within a few days. RNA samples were treated with RQ1 DNase (Promega, Madison, WI, USA) according to the instructions. The ratios at absorbencies of 260 to 280 and at 260 to 230 as well as gel electrophoresis were used to verify RNA quality. Poor quality samples were not used for subsequent experiments. RNA concentrations were determined using a NanoDrop 2000 (Thermo Scientific, Waltham, MA, USA).

For reverse transcription–PCR (RT–PCR), cDNA was made using the iScript™ cDNA Synthesis Kit (Bio-Rad, Hercules, CA, USA). For quantitative real-time PCR (qRT-PCR), cDNA synthesis and qRT–PCR were done simultaneously using a qScript™ One-Step qRT-PCR Kit (Quanta Biosciences, Gaithersburg, MD, USA). The samples were run on a LightCycler^®^ 480 Real Time-PCR System (Roche Applied Science, Basel, Switzerland). Each 6 μl reaction contained RNA at a final concentration of 2ng μl^–1^ and each primer at a concentration of 0.65 μM. Other components were included at the concentrations defined by the kit. Control reactions without reverse transcriptase were included and did not yield any detectable expression. Cycling conditions were 49 ºC for 10min, 95 ºC for 5min, and 45 cycles of 95 ºC for 5 s, 55 ºC for 15 s, and 72 ºC for 10 s. Melt curves were used to verify specificity. Four technical replicates were performed for each sample for each biological replicate, and three biological replicates were performed. LinRegPCR was used to analyse the data (Ramakers *et al.*, 2003).

The primers used to amplify *OCT1* were: 1F (5′ GTGGCTGTT CCTTCCACACT 3′), 1R (5′ AAAGGCCGTGACGAAAGTTA 3′), 2F (5′ CATCCTCGACAGCGTATGAC 3′), 2R (5′ CCTTC CTAACGCAACTAGCA 3′), 3F (5′ TTTGGTGTTGCATCAGTG CT 3′), and 3R (5′ CGGAGCGTTTCGAGTTTCT 3′). 4R (5′ CGTGAAATGCGTGTTGAAAG 3′) was used to detect possible transcript that extended into the T-DNA region. Primers 3F and 3R as well as 5′ CCTCTTGGATACGCGGTTT 3′ and 5′ CAAGA AGCCATCGAGGAGAC 3′ were used for *OCT1* qRT–PCR. Primers used to amplify the reference gene At1g58050 (Czechowski *et al.*, 2005) were: 5′ CCATTCTACTTTTTGGCGGCT 3′ and 5′ TCAATGGTAACTGATCCACTCTGATG 3′. This reference gene was chosen due to its consistent expression and its similar expression level to *OCT1* (Czechowski *et al.*, 2005)

### Transgenic constructs

Genomic DNA from Cvi and L*er* was used to amplify the region extending from 2079 bases before the start codon through to 417 bases after the stop codon of *OCT1*. This region was cloned in between the attL1 and attL2 sites in the Gateway entry vector pENTR/D-TOPO (Life Technologies, Madison, WI, USA). An LR reaction was performed to transfer this region into the binary vector pMDC99 (Curtis and Grossniklaus, 2003; Xu and Li, 2008). The constructs were sequenced and transformed into plants using the *Agrobacterium*-mediated floral dip method (Clough and Bent, 1998). T_3_ seedlings likely to all carry the transgene as determined by antibiotic resistance were used in all experiments (Harrison *et al.*, 2006).

## Results

### *Arabidopsis* accessions vary in their root length responses to cadaverine

**Fig. 1.**
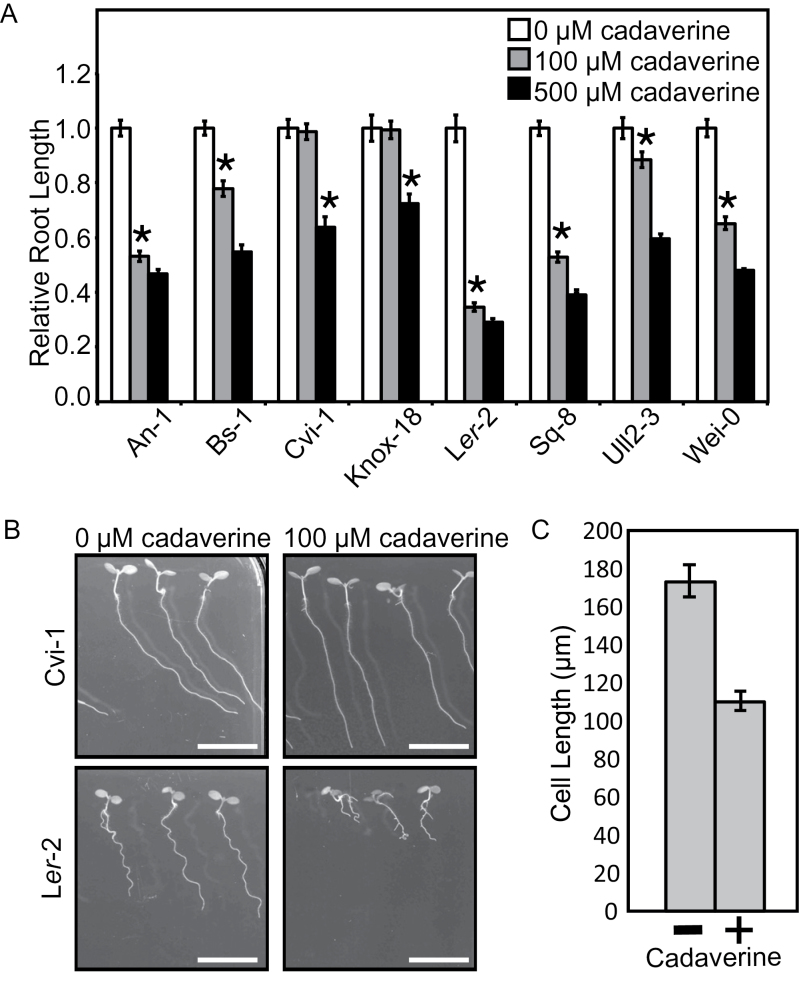
*Arabidopsis* accessions show varying root length inhibition responses to exogenous cadaverine. (A) Seedlings were grown vertically for 3 d and then at a 30 ° backward tilt for another 3 d. Average root length on control medium was set to 1. Absolute average root lengths on control medium were 1.4, 1.6, 2.5, 2.1, 2.1, 2.3, 2.0, and 2.3cm in the accessions as shown from left to right. The asterisk indicates the first cadaverine concentration corresponding to a significantly shorter root compared with control medium (*P*<0.05, Student’s *t*-test). Error bars indicate ±SE, and *n*=12–27. (B) Images of Cvi-1 (top) and L*er*-2 (bottom) seedlings growing on media without (left) and with (right) 100 μM cadaverine. The white scale bar is 1cm. (C) Average length of epidermal cells in the mature root zone from seedlings grown vertically for 7 d on either control medium or medium containing 100 μM cadaverine. Error bars indicate ±SE, and *n*=11–18.

As a first step toward identifying genes involved in root growth responses to exogenous cadaverine, seedlings of eight *Arabidopsis* accessions were grown on media containing 0, 100, and 500 μM cadaverine. A decrease in root length between control and increasing cadaverine treatments was observed for all accessions tested (Fig. 1A). Alterations in root waving and skewing and an increase in adventitious and lateral root emergence were also noticed in most accessions (Supplementary Fig. S1 at *JXB* online). In terms of root length, Cvi-1 and Knox-18 were some of the most resistant accessions to cadaverine, whereas L*er*-2 was one of the most sensitive (Fig. 1A, B).

To determine whether the effect of cadaverine on root length was due to reduced cell elongation or division, the length of the epidermal cells in the mature root zone of L*er* seedlings grown on media containing 0 μM or 100 μM cadaverine was measured. It was found that although L*er* roots were only ~35% as long on this concentration of cadaverine as on control medium (Fig. 1A), the average cell length of the seedlings on the medium containing cadaverine was ~64% of that of seedlings on control medium (Fig. 1C). This suggests that the reduction in root length is probably due to a combination of reduced cell elongation and division.

### A QTL on chromosome 1 affects root length responses to cadaverine

**Fig. 2.**
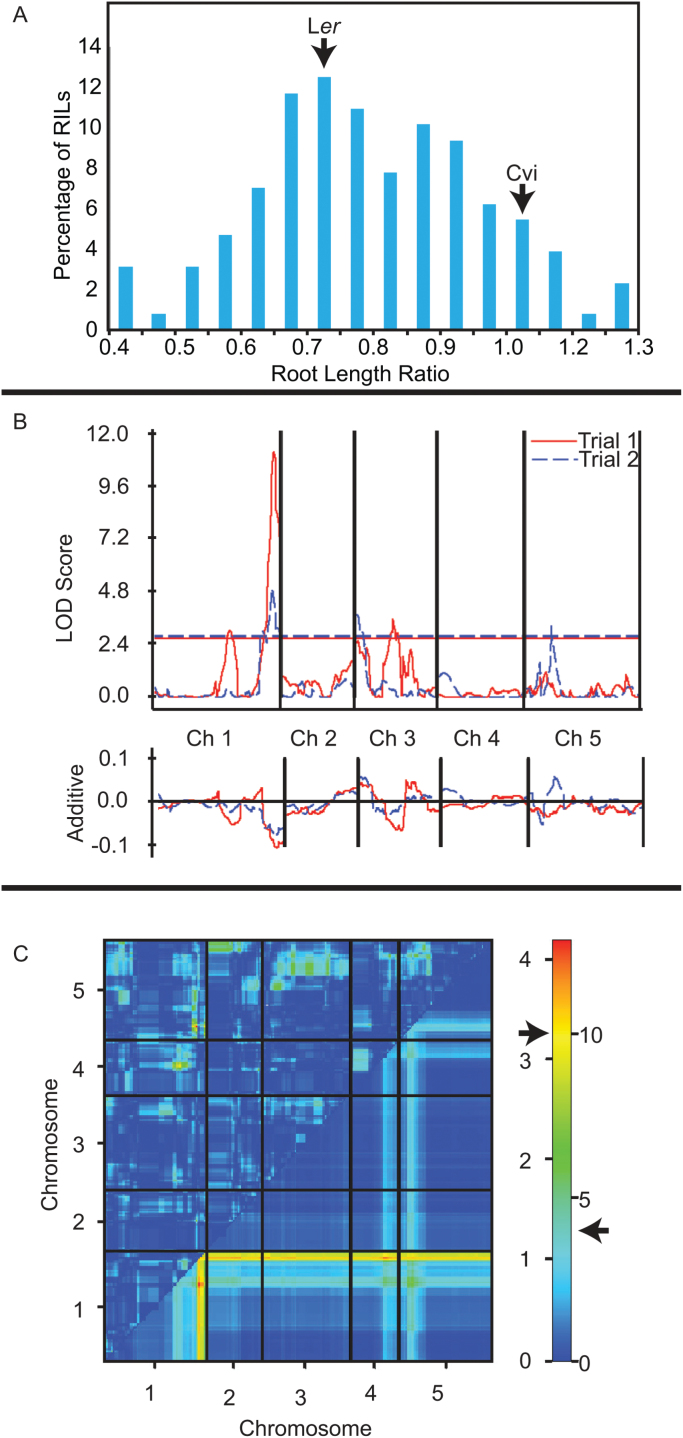
QTL analysis of root length responses to cadaverine in the Cvi/L*er* RIL population. (A) Histogram showing root length ratios for the RILs on 100 μM cadaverine compared with control medium. (B) Composite interval mapping analysis for the root length ratio. The top portion of the graph gives the LOD score across each chromosome. The LOD significance threshold is shown as a horizontal line across the graph. The bottom graph shows the additive value toward the phenotype of each genomic region with respect to the L*er* allele. (C) Two-dimensional QTL scan for the root length ratio from trial 1. The *x*- and *y*-axes represent positions along each chromosome. The region of the plot below the diagonal gives the additive QTL model, while the region above the diagonal shows epistatic interactions. A scale relating colour to LOD score is provided on the right of the heat map. Numbers on the left of this scale represent LOD scores for the epistatic portion of the plot, whereas those on the right represent scores for the additive model. The arrows indicate the significance thresholds for the epistatic (left) and additive (right) portions of the plot.

Due to the large difference between Cvi and L*er* in root growth responses to exogenous cadaverine, a QTL study was performed with a group of RILs created from the two accessions. Other pairs of accessions would have been equally interesting to use for QTL analysis but, at the time this experiment was initiated, the Cvi×L*er* population was the available population created from accessions with the most differing cadaverine responses. These RILs were previously characterized for genotypic contribution from the two parental lines along the five chromosomes (Alonso-Blanco *et al.*, 1998). For both the parental lines and the RILs, root growth on medium containing cadaverine compared with growth on control plates lacking this compound was quantified. RIL mean root length ratios showed transgression with respect to the parental means (Fig. 2A). Furthermore, growth on 0 μM and 50 μM cadaverine displayed broad sense heritability values of 0.46 and 0.56, respectively. Taken together, these results indicate that this population is well suited for QTL mapping of root growth responses to exogenous cadaverine.

Using both composite interval mapping and a 2D scan approach, a strong QTL for relative root growth response to cadaverine was identified at the end of chromosome 1 in two independent trials (Fig. 2B). The additive portion of the QTL graph (Fig. 2B, bottom) predicts that having a L*er* allele at this location results in a shorter root upon cadaverine treatment compared with a Cvi allele. No epistasis was observed between this QTL and other parts of the genome (Fig. 2C).

### Near isogenic lines confirm the chromosome 1 QTL

**Fig. 3.**
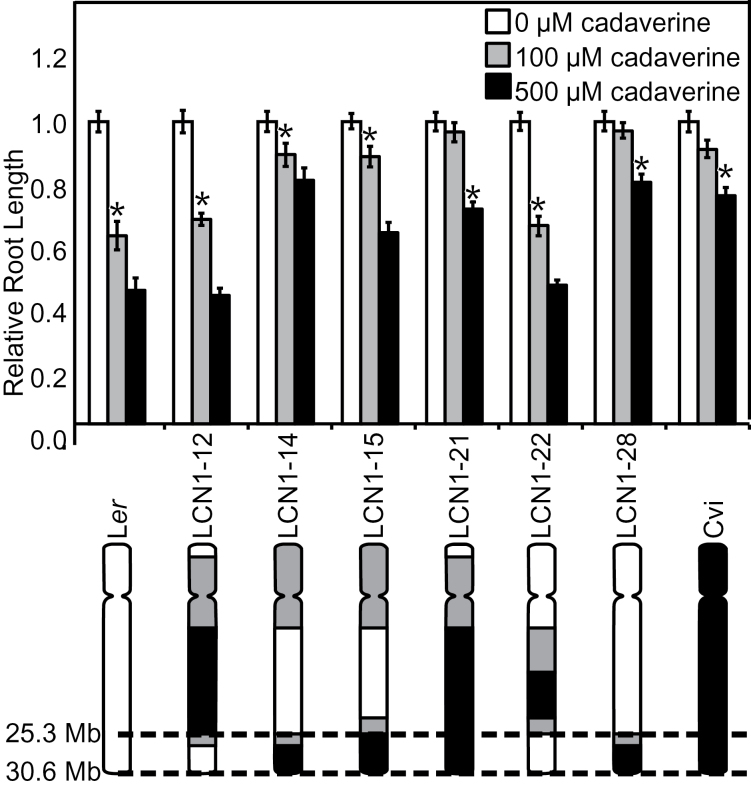
Cadaverine root length responses of chromosome 1 NILs and fine mapping. Relative root lengths on media containing 0, 100, and 500 μM cadaverine are shown. Plants were grown at a 30 ° backward tilt for 6 d. The average root length on control medium was set to 1. Absolute average root lengths on control medium were 1.0, 1.2, 1.2, 1.2, 1.1, 1.2, 1.1, and 1.1cm in the lines as shown from left to right. The asterisk indicates the first concentration on which the root length was significantly different from the length on control medium (*P*<0.05, Student’s *t*-test). Error bars indicate ±SE, and *n*=18–34. In the diagram of chromosome 1 NILs (bottom), white bars are L*er* chromosomal regions, black bars are Cvi regions, and grey bars indicate regions in which the breakpoint is undetermined. The parallel dashed lines flank the probable region of a locus controlling root length responses to cadaverine. Accession breakpoints were determined using primers based on polymorphisms described by Monsanto/CEREON between Columbia and L*er* (Jander *et al.*, 2002).

Six NILs with Cvi introgression of varying segments of chromosome 1 within an otherwise L*er* background (Keurentjes *et al.*, 2007) were used to confirm the root length QTL. The four LCN1 lines that contain a Cvi introgression at the end of chromosome 1 showed greater, more Cvi-like resistance to cadaverine than LCN1-12 and LCN1-22, the NILs that do not contain a Cvi introgression in this region (Fig. 3). The L*er* to Cvi breakpoints in these lines were fine-mapped, and the candidate region was narrowed to a 5.3Mb interval at the end of chromosome 1 (primers shown in Supplementary Table S1 at *JXB* online).

### Identification of candidate genes for the chromosome 1 QTL

Because many of the causative loci behind the QTL are due to differences in gene expression (Alonso-Blanco *et al.*, 2009), gene expression differences between Cvi and L*er* were examined in the candidate region using previously published microarray data (Vaughn and Masson, 2011). The primary data are publicly available at Gene Expression Omnibus (NCBI; accession number GSE28275). From these arrays, 19 probe sets representing genes located in the candidate chromosomal segment give hybridization signals that differ significantly (95% confidence interval) by ≥2-fold between Cvi and L*er* (Table 1). The largest predicted differentially expressed gene in this region was *OCT1*, which was estimated to show a 73-fold increase in Cvi compared with L*er*.

**Table 1.** Probe sets on chromosome 1 between 25.3Mb and 30.6Mb that show ≥2-fold differential expression between Cvi and L*er* at the 95% confidence level

Probe set ID	Locus ID	L*er* over Cvi fold change	Log_2_ expression level		Description
			L*er*	Cvi	
260097_at	AT1G73220	72.7 down	3.7	9.9	Organic cation transporter (OCT1)
264100_at	AT1G78970	48.5 down	1.9	7.5	Lupeol synthase (LUP1)
245215_at	AT1G67830	6.0 down	5.9	8.5	α-Fucosidase (FXG1)
245736_at	AT1G73330	5.1 down	10.9	13.3	Protease inhibitor (DR4)
259941_s_at	AT1G71280	4.4 down	5.2	7.4	DEAD/DEAH box helicase
260806_at	AT1G78260	2.9 down	9.4	11.0	RNA-binding protein
260401_at	AT1G69840	2.5 down	8.9	10.2	Unknown protein
264144_at	AT1G79320	2.2 down	7.4	8.5	Metacaspase (MC6)
259705_at	AT1G77450	2.0 up	12.1	11.1	NAC transcription factor (NAC032)
259977_at	AT1G76590	3.1 up	8.3	6.7	Zinc-binding protein
261901_at	AT1G80920	3.4 up	12.4	10.6	DNA-J protein (J8)
260411_at	AT1G69890	3.4 up	7.6	5.9	Unknown protein
264957_at	AT1G77000	3.7 up	10.1	8.2	F-box protein (SKP2B)
262362_at	AT1G72840	4.0 up	7.4	5.4	ATP-binding transmembrane receptor
260207_at	AT1G70730	4.7 up	10.8	8.6	Phosphoglucomutase
245731_at	AT1G73500	8.9 up	10.1	6.9	MAP kinase kinase (MKK9)
260101_at	AT1G73260	12.2 up	12.6	9.0	Trypsin protease inhibitor
259866_at	AT1G76640	14.3 up	7.4	3.6	Calmodulin-related protein
264720_at	AT1G70080	20.8 up	10.3	5.9	Terpene synthase

Abbreviations: NIL, near isogenic line; OCT, organic cation transporter; RIL, recombinant inbred line.

No other genes in Table 1 encode proteins that are directly relevant to polyamine metabolism or transport. Genes encoding a transglutaminase (*At1g69820*) and a cinnamyl alcohol dehydrogenase (*At1g72680*), both possibly linked to the formation of polyamine conjugates (Serafini-Fracassini and Del Duca, 2008; Fellenberg *et al.*, 2009), are found in this QTL interval; however, they do not show ≥2-fold expression differences between Cvi and L*er*.

**Fig. 4.**
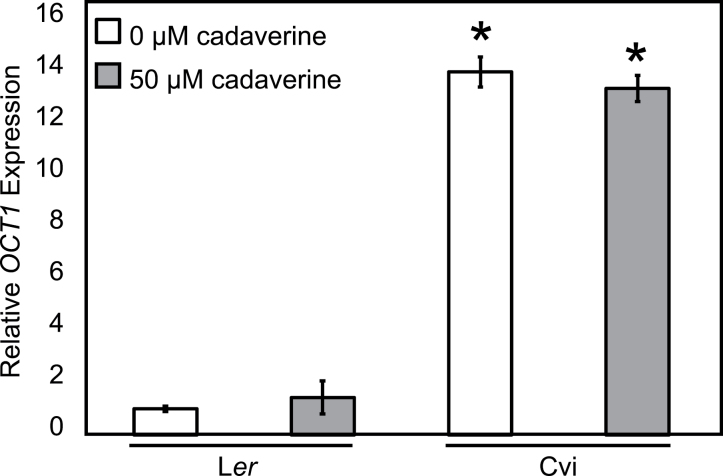
*OCT1* expression relative to the reference gene *At1G58050* is higher in Cvi roots than in L*er* roots. Seedlings were grown on 0 μM or 50 μM cadaverine. Error bars indicate ±SE among biological replicates, and *OCT1* expression for L*er* on control medium was set to 1. The asterisk indicates a significant difference in expression compared with L*er* for the given condition (*P*<0.05, Student’s *t*-test). Differences within each accession between cadaverine treatments are not significant.

*OCT1* from Cvi and L*er* genomic DNA was sequenced. No large insertions or deletions were found, and only one amino acid change was found: residue 431 in the second exon is serine in Cvi and alanine in L*er* (Supplementary Table S2 at *JXB* online). Analysing the 2kb of sequence upstream of the start codon revealed numerous single nucleotide polymorphisms and small insertions but no large insertions or deletions (Supplementary Table S2). Based on these data, it was hypothesized that *OCT1* expression differences contribute to root length responses to exogenous cadaverine. qRT-PCR data confirmed the microarray result and showed that *OCT1* expression was ~14 times as high in Cvi roots as in L*er* roots (Fig. 4). Cadaverine treatment did not significantly change *OCT1* expression in either accession (Fig. 4).

### *oct1-1* mutants express a truncated *OCT1* transcript

To evaluate a role for *OCT1* in root length responses to cadaverine, the *oct1-1* mutant, which was previously found to carry a single T-DNA insertion near the 3’ end of the gene, was obtained; both ends of the insertion were characterized (Lelandais-Brière *et al.*, 2007). This mutant was also reported to not express any *OCT1* RNA, as determined by RT–PCR using primers aligning on each side of the intron (Lelandais-Brière *et al.*, 2007). To confirm this result, RT–PCR was performed on *oct1-1* and (Wassilewskija) WS wild-type root RNA samples. As expected, PCR products were obtained from WS wild-type cDNA samples of the expected sizes using multiple primer sets (1F/1R, 2F/2R, and 3F/3R) (Supplementary Fig. S2 at *JXB* online). Also, no transcript was observed for *oct1-1* mutants using primer sets near the 3′ end of the gene (2F/2R, 3F/3R, and 2F/4R) (Supplementary Fig. S2).

However, it was found that *oct1-1* plants do express a partial *OCT1* transcript using primers on each side of the intron (1F/1R) (Supplementary Fig. S2 at *JXB* online). This product size corresponds to approximately the predicted length if the intron is not spliced out of the RNA (238 bases instead of 135 bases), which would result in a premature stop codon in the intron. No product for any primer set was detected in control reactions without reverse transcriptase, which confirms the lack of genomic DNA contamination (Supplementary Fig. S2). Therefore, *oct1-1* produces a truncated *OCT1* transcript that is highly unlikely to encode a functional protein.

### *oct1-1* shows increased cadaverine sensitivity

**Fig. 5.**
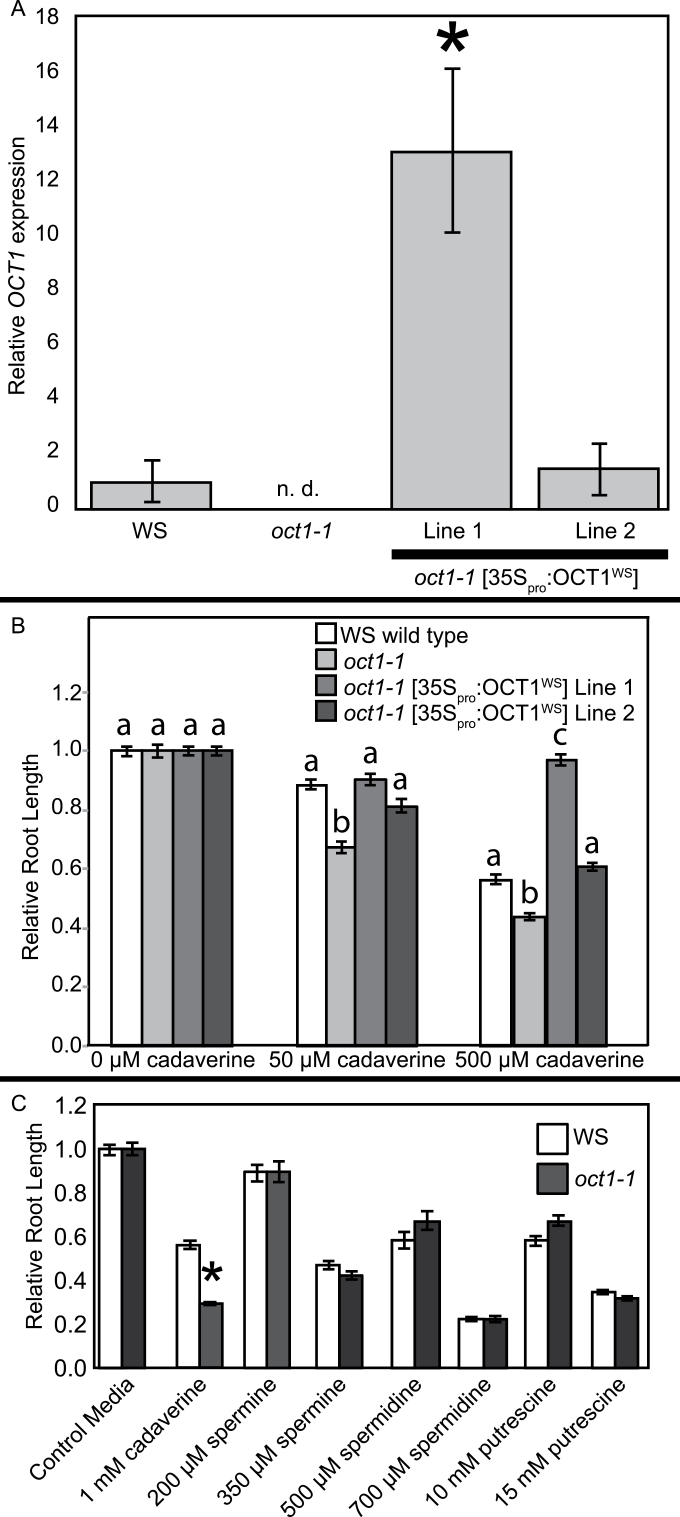
*oct1-1* roots are more sensitive to exogenous cadaverine compared with wild-type plants but respond similarly to putrescine-derived polyamines. (A) *OCT1* expression relative to the reference gene *At1G58050* is shown. Error bars indicate ±SE among biological replicates, and *OCT1* expression for the WS wild type on control medium was set to 1. The asterisk indicates a significant difference in expression compared with the WS wild type (*P*<0.05, Student’s *t*-test). The abbreviation n.d. indicates that expression was not detected. (B) Plants were grown for 6 d at a 30 ° backward tilt on media containing 0, 50, and 500 μM cadaverine. Root tips were marked after 3 d of growth, and the root growth over the following 3 d was measured for each treatment. Average root growth over the final 3 d on control medium was 1.2cm for the wild type, 1.4cm for *oct1-1*, 1.3cm for rescue line 1, and 1.4cm for rescue line 2; these lengths were set to 1. For each cadaverine concentration, the letters indicate significant differences in relative root length (*P*<0.01, pairwise Student’s *t*-tests). *n*=37–68, and error bars indicate ±SE. (C) Plants were grown for 6 d at a 30 ° backward tilt on media containing various concentrations of polyamines as indicated. Average root length on control medium was 1.3cm for the wild type and 1.4cm for oct1-1; these lengths were set to 1. *n*=33–44, and error bars indicate ±SE. No significant root length differences were observed for any treatment other than cadaverine (*P*<0.05, Student’s *t*-test).

If L*er* roots have higher sensitivity to cadaverine compared with Cvi roots because they have lower *OCT1* expression, then *oct1* mutant roots should also respond more strongly to cadaverine than wild-type plants. To test this, WS wild type, *oct1-1*, and two independent transgenic rescue lines (*oct1-1* [35S_pro_:OCT1^WS^]) were grown on control medium and medium supplemented with cadaverine. Rescue line 1 had significantly higher *OCT1* expression compared with the WS wild type, whereas rescue line 2 had a similar level of *OCT1* expression (Fig. 5A). It was found that all of these plants had the same root length on control medium lacking cadaverine, but *oct1-1* mutant roots were significantly shorter than wild-type roots on media containing 50 μM and 500 μM cadaverine (Fig. 5B). Robust root growth on medium containing cadaverine was restored in both independent transgenic rescue lines. Rescue line 2 showed a wild-type-like response to cadaverine, and rescue line 1 showed increased resistance to cadaverine (Fig. 5B). These results suggest that reduced *OCT1* expression confers increased cadaverine sensitivity.

The WS wild type and *oct1-1* had similar root lengths on media containing the polyamines putrescine, spermidine, and spermine (Fig. 5C), which shows that this sensitivity is specific to cadaverine. Polyamines have been shown to shorten root growth through the generation of H_2_O_2_ by diamine and polyamine oxidases, which promotes premature cell differentiation and programmed cell death (Tisi *et al.*, 2011). However, because the cadaverine-hypersensitive response of *oct1-1* was specific to cadaverine (Fig. 5C), OCT1 probably functions in a different aspect of polyamine-mediated root growth regulation.

### The expression level of *OCT1*, not its accession origin, correlates with cadaverine sensitivity

**Fig. 6.**
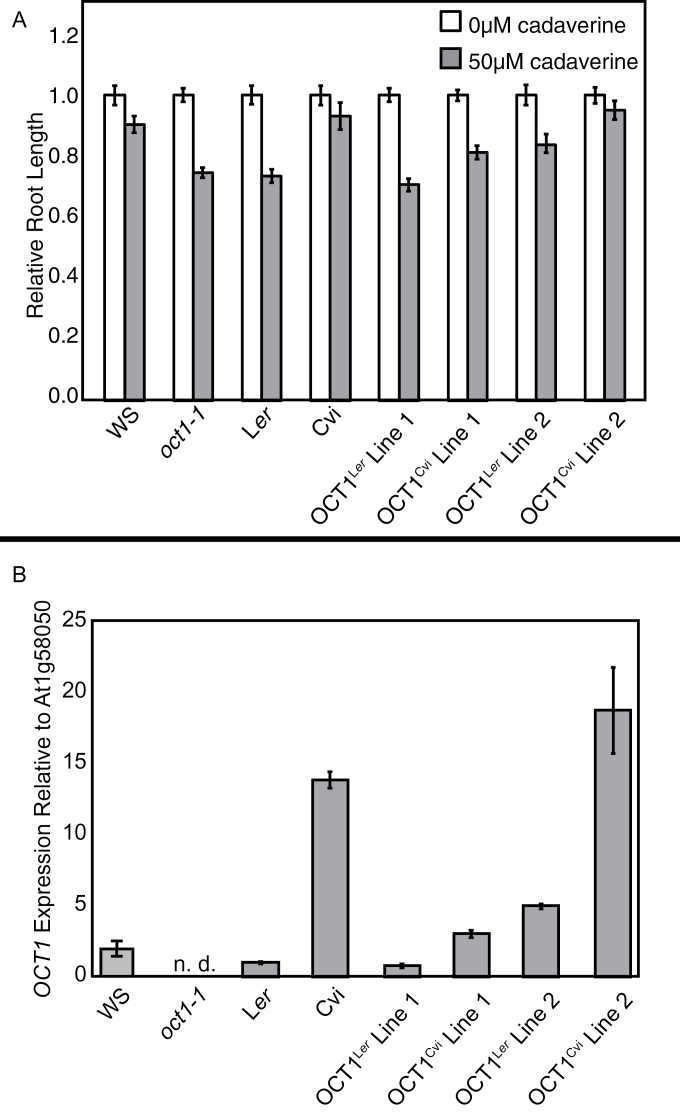
Higher *OCT1* expression correlates with cadaverine resistance. OCT1^Cvi^ and OCT1^L*er*^ indicate *oct1-1* [OCT1_pro_:OCT1^Cvi^] and *oct1-1* [OCT1_pro_:OCT1^L*er*^], respectively. (A) Relative root lengths of plants on media containing 0 μM and 50 μM cadaverine. Plants were grown for 6 d at a 30 ° backward tilt. Average root length on control medium was set to 1. Absolute average root length on control medium was 2.2cm for Cvi, 1.9cm for L*er*, 2.0cm for WS, 2.0cm for *oct1-1*, and 1.9, 2.0, 1.7, and 2.1cm for the transgenic lines as shown from left to right. Error bars indicate ±SE, and *n*=17–24. (B) Root *OCT1* expression determined by qRT-PCR is shown relative to the reference gene *At1G58050*. *OCT1* expression in L*er* was set to 1. Error bars indicate ±SE among the biological replicates. The abbreviation n.d. indicates that expression was not detected.

To determine if differences in the *OCT1* sequence between Cvi and L*er* or only *OCT1* expression levels correlated with the cadaverine response, *OCT1* was cloned from Cvi and L*er*, and these constructs were introduced into *oct1-1* mutants. These transgenic lines were then tested on media containing 0 μM and 50 μM cadaverine, and their relative root lengths were measured. A range of cadaverine resistance levels that did not correlate with the accession source of the transgene was found (Fig. 6A).

Because the insertion location can affect transgene expression, it was predicted that the *OCT1* expression levels would correlate with cadaverine sensitivity. To test this, *OCT1* expression was measured in four of these lines by qRT-PCR. As expected, a range of *OCT1* expression levels was found in the transgenic lines regardless of whether the *OCT1* untranslated and coding regions came from Cvi or L*er*. In general, lines with increased resistance to cadaverine had higher *OCT1* expression (Fig. 6).

### Genes other than *OCT1* probably also contribute to the cadaverine response

The data suggest that differences in *OCT1* expression contribute significantly to the different cadaverine root length responses in Cvi and L*er*. To determine if differences in *OCT1* expression among other natural accessions also correlate with variability in cadaverine sensitivity, *OCT1* expression was measured in two other accessions, one resistant to cadaverine (Knox-18) and one sensitive to cadaverine (Sq-8). It was found that both lines had higher *OCT1* expression than L*er* even though only Knox-18 was more resistant to cadaverine than L*er*. Furthermore, Sq-8 displayed higher *OCT1* expression than Knox-18 despite being more sensitive to cadaverine (Supplementary Fig. S3 at *JXB* online). The *OCT1* sequences of the eight natural accessions initially selected for the analysis of cadaverine response were also compared. Polymorphisms shared among the three most resistant accessions (Cvi-1, Knox-18, and Ull2-3) or the three most sensitive accessions (An-1, L*er*-2, and Sq-8) were not seen (Supplementary Table S2). Together, these data suggest that factors other than *OCT1* expression may also contribute significantly to differential cadaverine sensitivity among these accessions.

Ectopic copies of *OCT1* derived from Cvi or L*er* were also introduced into Columbia and L*er* wild-type plants. L*er* root length is highly sensitive to cadaverine, whereas Columbia root length is less sensitive to it. In general, the Columbia plants showed a range of responses to cadaverine regardless of the accession from which the transgene was derived (Supplementary Fig. S4 at *JXB* online). This result is similar to that obtained when these constructs were transformed into the *oct1-1* mutant (Fig. 6). In contrast, when these constructs were transformed into L*er* plants, the tested lines generally did not show increased resistance to cadaverine (Supplementary Fig. S4). Therefore, the ability of *OCT1* to affect the cadaverine response may depend on the genetic background.

## Discussion

It was found that *Arabidopsis* roots exhibited varying responses to exogenously applied cadaverine depending on the accession. In general, when cadaverine concentrations increased, a reduction in root length as well as alterations in skewing, lateral root branching, and root waving were seen. The reduction in root length is probably due to a combination of reduced cell division and expansion. A major QTL was identified on chromosome 1 that at least partially explains the root length difference in response to cadaverine between Cvi and L*er*. By comparing phenotypes and gene expression in natural accessions, a mutant, and transgenic lines, *OCT1* was identified as a key gene involved in this response. Restoring *OCT1* expression in the mutant background resulted in a positive correlation between *OCT1* expression levels and cadaverine resistance. Because the *OCT1* expression levels in other natural accessions do not correlate well with cadaverine resistance, other genes are also likely to contribute to the variation of this trait between *Arabidopsis* accessions. These genes could contribute to endogenous cadaverine homeostasis, transport, and/or response. Unfortunately, the levels of endogenous cadaverine in *Arabidopsis* seedling roots and shoots were below the level of detection using HPLC methods, preventing a distinction from being made between these possibilities.

OCT1 encodes an organic cation transporter. In animals, OCT proteins take up a wide variety of endogenous compounds and xenobiotics, and, therefore, they affect substrate absorption, distribution, and excretion in many tissues. Most are polyspecific, and transport can occur in either direction (for a review, see Roth *et al.*, 2012). *Arabidopsis* OCT1 is one of six OCT-like proteins in *Arabidopsis* (Lelandais-Brière *et al.*, 2007). It was previously reported to localize to the plasma membrane, and promoter–β-glucuronidase (GUS) fusions showed activity in the vasculature of many organs including roots (Lelandais-Brière *et al.*, 2007; Küfner and Koch, 2008). Because the other six OCT-like proteins localize to the tonoplast rather than the plasma membrane, it is likely that OCT1 has a different function.

The present work expands on these studies and suggests a role for OCT1 in affecting root growth responses to cadaverine. Future experiments will be aimed at clarifying the mechanism by which this occurs. One possibility is that OCT1 transports cadaverine away from elongating root cells and through the vasculature, causing it to accumulate in aerial tissues. Alternatively, cadaverine may alter the transport of another molecule through the membranes. Polyamines block many kinds of channels in multiple organisms. For example, spermine and spermidine directly block fast vacuolar cation channels in barley (Bruggemann *et al.*, 1998), and all natural polyamines including cadaverine can regulate cation channel activity through cytoplasmic pathways (Liu *et al.*, 2000; Shabala *et al.*, 2007).

It has been demonstrated that exogenous cadaverine affects *Arabidopsis* root morphology and that there is natural variation in this response due in part to varying expression levels of *OCT1*. This work has potential implications for better understanding how polyamines contribute to development and mediate stress responses.

## Supplementary data

Supplementary data are available at *JXB* online.

Figure S1. *Arabidopsis* natural accessions show varying root growth behaviours in response to cadaverine.

Figure S2. *oct1-1* produces a truncated transcript.

Figure S3. *OCT1* expression does not correlate with cadaverine sensitivity in all natural accessions.

Figure S4. *OCT1* expression results in varying degrees of cadaverine sensitivity in Col and L*er*.

Table S1. Primers for chromosome 1 fine mapping.

Table S2. Comparison of *OCT1* sequences from eight *Arabidopsis* natural accessions.

Supplementary Data
