# Supplementary material for: Natural variation in the expression of ORGANIC CATION TRANSPORTER 1 affects root length responses to cadaverine in Arabidopsis
Source: J Exp Bot. 2014 Nov 16;66(3):853–62. doi: 10.1093/jxb/eru444 (PMC4321547; doi:10.1093/jxb/eru444)
Supplement: Supplementary Data [file supp_66_3_853__index.html]

Natural variation in the expression of ORGANIC CATION TRANSPORTER 1 affects root length responses to cadaverine in Arabidopsis — Natural variation in the expression of ORGANIC CATION TRANSPORTER 1 affects root length responses to cadaverine in Arabidopsis — Supplementary Data 

# Natural variation in the expression of *ORGANIC CATION TRANSPORTER 1* affects root length responses to cadaverine in *Arabidopsis*

## Supplementary Data

Data files

**Files in this Data Supplement:**

- Supplementary Data - Supplementary Data
