# Supplementary material for: Natural variation in the expression of ORGANIC CATION TRANSPORTER 1 affects root length responses to cadaverine in Arabidopsis
Source: J Exp Bot. 2014 Nov 16;66(3):853–62. doi: 10.1093/jxb/eru444 (PMC4321547; doi:10.1093/jxb/eru444)
Supplement: Supplementary Data [file supp_eru444_jexbot126433_file001.pdf]

Table S1. Primers for chromosome 1 fine mapping.

Forward Primer	Reverse Primer	Forward Primer Start (bp)[1]	Cvi Product Size (bp)	L*er* Product Size (bp)
gtgagctcttcagcagttagcac	ggtcagattgttgttaggtg	11016704	253	156
tgctgacattctccaaaacct	gcatggcttatccatttggt	17339898	570	533
aagcaaaaggtgaacacagg	ttcgattcgatcataagcatt	18754107	288	239
aaaacaatccatcttacacacca	tttgcgcatagctacactctaca	20072062	272	246
cttgcattgttgagtccttga	aagcgaaccccttcgataat	23211588	1250	360
tcatttctaagttgagccacga	tgcttcatatgtagaagccaataa	25261117	262	220
tcaatgactcaagcagctcaa	cagcaaaatgccttgtcgta	26460199	600	568
caatagaatttggctgccgtgcca	attacgtgcctctcttgtccgcta	27355508	263	218
gtgacgtgatctttcacacttttt	gtcaccatcgccactaacac	29782305	287	254

[1]TAIR build 9

Table S2. Comparison of *OCT1* sequences from eight Arabidopsis natural accessions. Cvi-1 and L*er*-2 sequences were obtained by Sanger sequencing. An-1, Bs-1, Knox-18, Sq-8, and Wei-0 sequences were obtained from Schmitz et al., 2013, and the Ull2-3 sequence was obtained from Long et al., 2013. The shaded area represents the protein coding region, and amino acid changes are shown in parenthesis.

Base	Col-0	An-1	Bs-1	Cvi-1	Knox-18	Ler-2	Sq-8	Ull2-3	Wei-0
27536383	A					T			
27536384	C					T			
27536562	C		T		T		T		
27536649	A	C						C	
27536720	G		A		A				
27536725	C		T		T				
27536766	C	T	T		T			T	
27536862	T	C	C					C	
27536894	G		A		A				
27536907	T	A	A						
27536933	C		A		A			A	
27536939	A		C		C				
27536947	C				T				
27536951	A				T				
27536952	A			T	T				
27536983	C			T		T			
27536024	A	Del.	Del.	Del.	Del.	Del.			
27536044	C	G		G		G		G	
27536065	C			G					
27536072	A			G					
27536121	G		T		T				
27536126	A						Del.		
27536132	C		Del.		Del.				
27536193	G	T						T	
27536212	A			T					
27536234	C	A	A	A	A	A	A	A	A
27536355	A			Ins. TATT					
27536429	T			Ins. T					
27536443	C	T		T		T		T	
27536457	A	T		T		T		T	
27536465	T	G		G		G		G	
27536505	T					A			
27536523	T	A		A				A	
27536653	C			A		A			
27536656	G			T					
27536681	T	G						G	
27536734	C	G						G	
27536085	T			A					
27536847	T	A	A	A	A	A	A	A	A
27536898	T	Del.							
27536917	A	T							

Base	Col-0	An-1	Bs-1	Cvi-1	Knox-18	Ler-2	Sq-8	Ull2-3	Wei-0
27536919	A	T							
27536922	T	A							
27536025	A					Del.			
27536141	A			G					
27536195	A			Ins. TA					
27536217	G	T		T				T	
27536264	T				C				
27536689	T		C						
27536753	G				A<br>(V to I)				
27536058	T		C<br>(V to A)						
27536705	C			T					
27536738	C	A<br>(R to S)						A<br>(R to S)	
27536781	G			T<br>(A to S)					
27536972	C					T			
27536995	G	A						A	
27536046	C								T
27536119	G	C		C		C		C	
27536264	G			Del.					
27536324	A				C				
27536361	G		T						
27536544	G						C		
27536843	G						A		A
27536955	A		G						

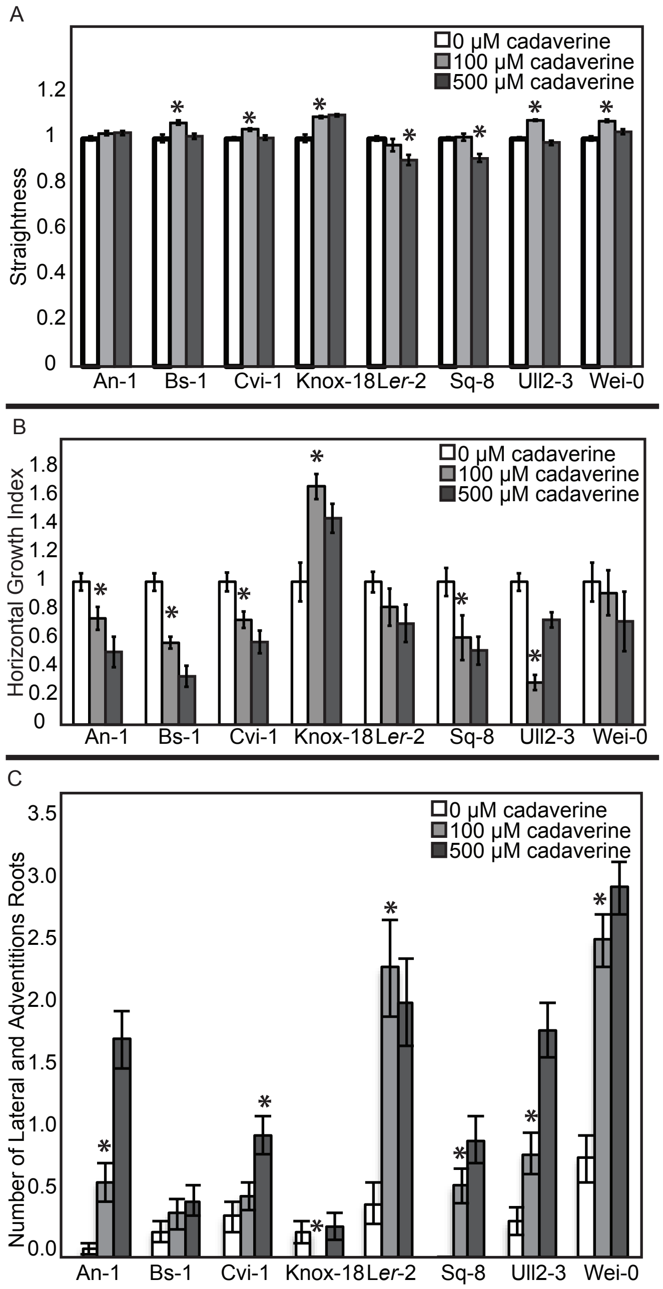

Figure S1. Arabidopsis natural accessions show varying root growth behaviors in response to cadaverine. Error bars indicate ± standard error, and n = 12–27. An asterisk indicates the first cadaverine concentration on which a significantly different growth behavior was observed ($p < 0.05$, Student's T-Test). **(A)** Straightness was calculated by dividing the direct distance from the base of the hypocotyl to the root tip by the total length of the root and setting this value on control medium to 1. **(B)** Horizontal growth index was calculated by dividing the distance traveled by the root along the x-axis by the total length of the root and setting this value on control medium to 1. **(C)** The average number of lateral and adventitious roots per seedling is shown.

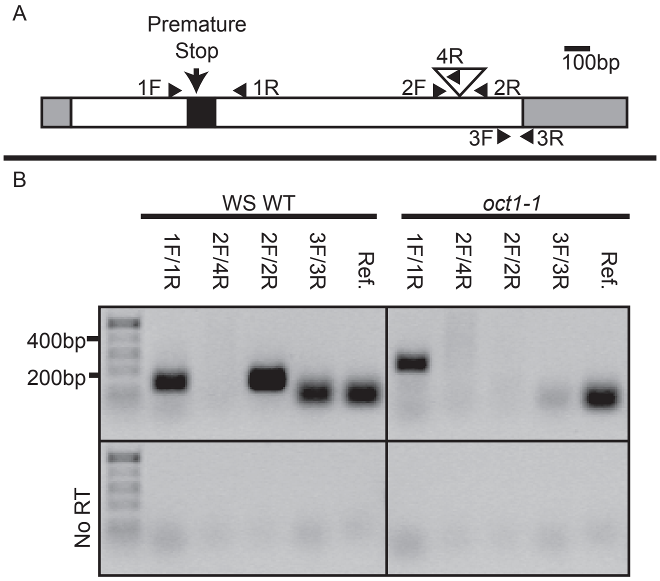

Figure S2. *oct1-1* produces a truncated transcript. **(A)** The gene diagram shows the locations of the primers used to characterize *oct1-1*. The arrow marks the position of the likely premature stop codon in *oct1-1*. White boxes indicate exons, the black box indicates the intron, grey boxes mark the 5' and 3' untranslated regions, and the triangle shows the location of the T-DNA insertion. **(B)** Gel electrophoresis image showing RT-PCR products from WS wild type and *oct1-1* root samples (top) as well as the corresponding controls done without reverse transcriptase (bottom) using primers aligning to various positions along the *OCT1* transcript or the reference gene *At1G58050*. In both cases, the left lane contained a DNA molecular weight marker (Hi-Lo DNA Marker, Minnesota Molecular, Minneapolis, MN).

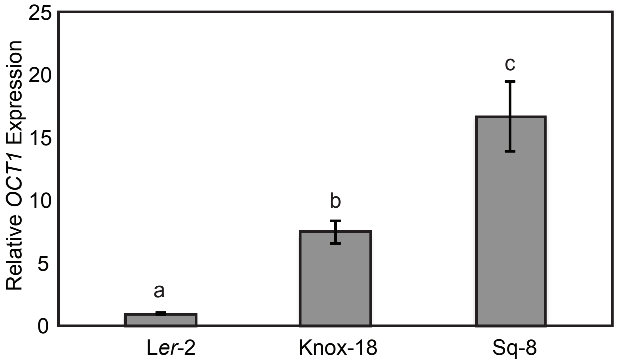

Figure S3. *OCT1* expression does not correlate with cadaverine sensitivity in all natural accessions. *OCT1* expression is shown relative to the reference gene *At1G58050*. Error bars indicate ± standard error among biological replicates, and *OCT1* expression for L*er* was set to 1. The letters indicate significant differences in expression ($p < 0.05$, pairwise Student's T-Tests).

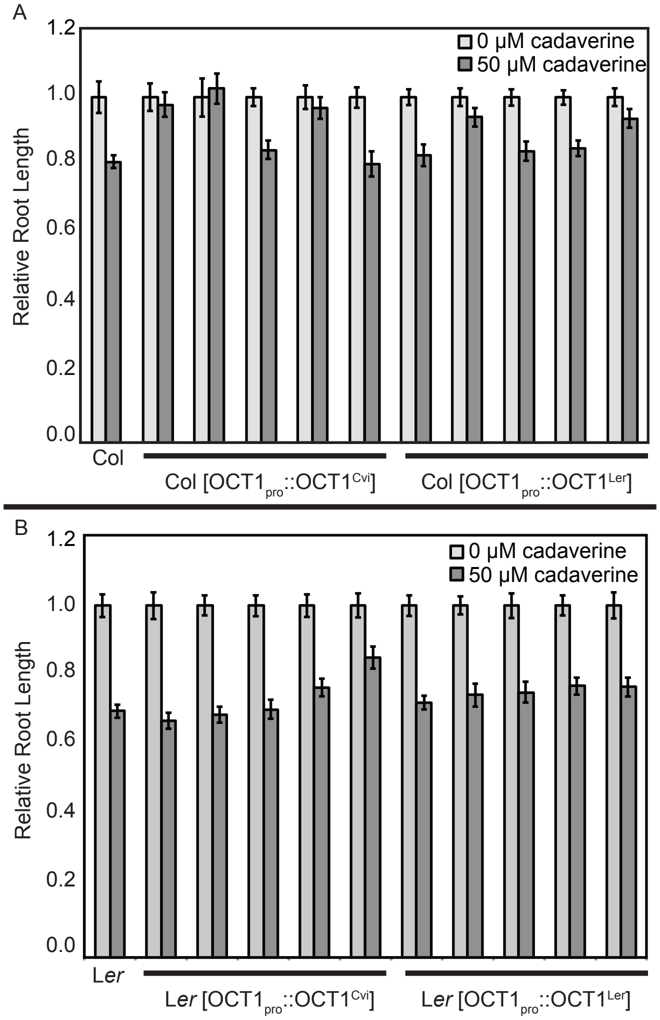

Figure S4. *OCT1* expression results in varying degrees of cadaverine sensitivity in Col and L*er*. Relative root lengths of plants on media containing 0 and 50 μM cadaverine. In each panel, the non-transformed accession is shown on the far left, and five independent lines with each transgene are shown to the right. Plants were grown for six days at a 30° tilt backward. Average root length on control medium was set to 1. In panel A, absolute average root length on control medium was 2.1 for Columbia and 1.9, 2.0 1.9, 1.7, 1.9, 2.1, 2.0, 2.2, 2.1, and 2.0 cm for the transgenic lines as shown from left to right. In panel B, absolute average root length on control medium was 2.3 cm for L*er* and 2.0, 2.0, 1.9, 2.2, 2.1, 2.1, 1.9, 2.0, 1.8, and 2.1 cm for the transgenic lines as shown from left to right. Error bars indicate ± standard error, and n = 13–34.
